# Using thoracic ultrasound to accurately assess pneumothorax progression during positive pressure ventilation: a comparison with computed tomography

**DOI:** 10.1186/1757-7241-21-S1-S3

**Published:** 2013-05-28

**Authors:** NP Oveland, HM Lossius, K Wemmelund, PJ Stokkeland, L Knudsen, E Sloth

**Affiliations:** 1 Department of Research and Development, Norwegian Air Ambulance Foundation, Droebak, Norway; 2 Department of Anesthesiology and Intensive Care, Stavanger University Hospital, Stavanger, Norway; 3 Department of Surgical Sciences, University of Bergen, Bergen, Norway; 4 Faculty of Health Sciences, Institute of Clinical Medicine, Aarhus University, Aarhus, Denmark; 5 Department of Radiology, Stavanger University Hospital, Stavanger, Norway; 6 Department of Anesthesiology and Intensive Care, Aarhus University Hospital, Aarhus, Denmark

## Objectives

While thoracic ultrasonography accurately determines the size and extent of occult pneumothoraces (PTXs) in spontaneously breathing patients, there is uncertainty about patients receiving positive pressure ventilation. We compared the lung point (i.e. the area where the collapsed lung still adheres to the inside of the chest wall) using the two modalities ultrasound (US) and computed tomography (CT), to determine whether US can reliably be used to assess PTX progression in a positive pressure ventilated porcine model.

## Methods

Air was introduced in incremental steps into five hemithoraces in three intubated porcine models. The lung point was identified on US imaging and referenced against the lateral limit of the intrapleural air space identified on the CT. The distance from the sternum to the lung point (S-LP) was measured on the CT scans and correlated to the insufflated air volume.

## Results

The mean total difference between the 131 US and CT lung points was 6.8 mm (standard deviation ± 7.1 mm and range 0.0-29.3 mm). A mixed-model regression analysis showed a linear relationship between the S-LP distances and the PTX volume (p <0.001).

## Conclusions

In an experimental porcine model, we found a linear relation between the PTX size and the lateral position of the lung point. The accuracy of thoracic US for identifying the lung point (and thus the PTX extent) was comparable to that of CT imaging. These clinically relevant results suggest that US may be safe and accurate in monitoring PTX progression during positive pressure ventilation.

